# Reviving the Transcriptome Studies: An Insight Into the Emergence of Single-Molecule Transcriptome Sequencing

**DOI:** 10.3389/fgene.2019.00384

**Published:** 2019-04-26

**Authors:** Bo Wang, Vivek Kumar, Andrew Olson, Doreen Ware

**Affiliations:** ^1^Cold Spring Harbor Laboratory, Cold Spring Harbor, NY, United States; ^2^USDA-ARS Robert W. Holley Center for Agriculture and Health, Ithaca, NY, United States

**Keywords:** transcriptomics, RNA-Seq, Iso-Seq, single-molecule transcriptome sequencing, alternative splicing, isoforms

## Abstract

Advances in transcriptomics have provided an exceptional opportunity to study functional implications of the genetic variability. Technologies such as RNA-Seq have emerged as state-of-the-art techniques for transcriptome analysis that take advantage of high-throughput next-generation sequencing. However, similar to their predecessors, these approaches continue to impose major challenges on full-length transcript structure identification, primarily due to inherent limitations of read length. With the development of single-molecule sequencing (SMS) from PacBio, a growing number of studies on the transcriptome of different organisms have been reported. SMS has emerged as advantageous for comprehensive genome annotation including identification of novel genes/isoforms, long non-coding RNAs and fusion transcripts. This approach can be used across a broad spectrum of species to better interpret the coding information of the genome, and facilitate the biological function study. We provide an overview of SMS platform and its diverse applications in various biological studies, and our perspective on the challenges associated with the transcriptome studies.

## Introduction

The last few decades have witnessed an explosive growth in the genomic sequencing technologies (Shendure et al., 2017). As a result of increased throughput, higher accuracies and lower costs, there has been an exponential growth in genomic sequence databases over the last two decades (Lathe et al., 2008; Heather and Chain, 2016; Levy and Myers, 2016; Ardui et al., 2018; Karsch-Mizrachi et al., 2018). However, a major challenge in the molecular biology continues to be the complex mapping of the same genome to diverse phenotypes in different tissue types, development stages and environmental conditions. A better understanding of the transcripts and expression of gene regulation is not only non-trivial but lies at the heart of this challenge. Transcriptomics offers important insights on gene structure, expression, and regulation and has been widely studied in many organisms (Jain, 2012; Casamassimi et al., 2017; Lowe et al., 2017). The transcriptomics studies have advanced considerably because of the explosive growth in the underlying sequencing technology (Abdel-Ghany et al., 2016; Wang et al., 2016).

Our objective here is to outline the current standards and resources for the platform and the bioinformatics approaches underlying the transcript profiling. We also aim to provide an overview of the single-molecule transcriptome sequencing workflow, particularly PacBio Iso-Seq, and briefly discuss various tools at different stages of the workflow. While we cover the broader technology landscape in this paper, we do not aim to provide an exhaustive compilation of resources or software tools or a highlight of the select tools. We finally conclude with a brief discussion of the opportunities as well as challenges associated with long read transcript profiling as compared to traditional short read techniques such as RNA-Seq.

## Evolution of Sequencing Technologies

First generation sequencing is primarily represented by the DNA sequencing approach pioneered by Sanger and Coulson, 1975 and is based on the selective incorporation of chain-terminating dideoxynucleotides by DNA polymerase during *in vitro* replication of DNA (Sanger and Coulson, 1975, Sanger et al., 1977). Another DNA sequencing approach was developed a year later by Maxam and Gilbert (1977) which was based on partial chemical modification of DNA specific to nucleotide bases and a subsequent cleavage of the DNA backbone at sites adjacent to the modified nucleotides. Unlike Sanger approach which required cloning to generate single strand DNA, Maxam–Gilbert sequencing was advantageous since it could directly use the purified DNA (Saccone and Pesole, 2003). However, Sanger’s chain termination method proved to be relatively easier to scale with the improvement of the chain-termination method and was widely used for next three decades including for the first draft of the Human Genome project. While it could sequence DNA fragments as long as 1 kb with a high raw read accuracy, it was limited by the low throughput and high cost (Schloss, 2008).

Second generation of sequencing (SGS) alternatively referred as next generation sequencing (NGS) technology, originated in mid 2000s to support massively parallel sequencing of hundreds of thousands of short DNA strands that are anchored and read through multiple “wash and scan” cycles (Moorthie et al., 2011; Goodwin et al., 2016). For example, Illumina HiSeq platforms can generate upward of 5 billion reads and 1500 Gb per run. Also, this approach is able to generate high read accuracy with much lower cost. However, the reads are generally limited in length to couple of 100 s of bases because of incremental errors introduced by the “wash and scan” cycles since the likelihood of incorporation of an extra base or failure of incorporation of a base increases during each step (Whiteford et al., 2009). Another limitation of this approach is amplification bias and the template sequence errors contributed by the polymerase chain reaction (PCR) amplification step. Admittedly, NGS has many applications in biological studies, such as DNA-sequencing to assemble a previously unknown genome, and RNA-sequencing to analyze gene expression and to identify the regions of DNA or RNA binding proteins. One of the most important applications of NGS is to identify mutations, including single nucleotide polymorphisms (SNP), small insertions/deletions (INDELs), structural variations, e.g., translocations, inversions, and copy number variations (CNV) (Zhang et al., 2011; Bahassi el and Stambrook, 2014; Wadapurkar and Vyas, 2018).

There are a number of different sequencing approaches that constitute the third generation of sequencing (TGS) paradigm, however, they are primarily distinguished from previous generations in their focus on uninterrupted sequencing of a single DNA or RNA molecule (not an ensemble). This makes them highly preferable for a number of use cases such as *de novo* assembly, improved genome annotations, and epigenome characterization (Blow et al., 2016; Seo et al., 2016; Jiao et al., 2017). One of the most significant among these approaches is the Single Molecule Real-Time (SMRT) sequencing pioneered by Pacific Biosciences. It uses nanoscale optical waveguide, more specifically zero-mode waveguide (ZMW) technology to be able to directly observe a single DNA polymerase molecule synthesizing a DNA strand. While it is in principle a sequencing by synthesis like Illumina, it does not depend on the “scan and wash” cycles and is therefore able to sequence very long reads largely limited in length by the chemistry of the DNA polymerase and not the underlying technology. As a result, it is possible to get reads of maximum length more than 80 kb and average length above 20 kb (Badouin et al., 2017; Jiao and Schneeberger, 2017). Also, it does not suffer from the amplification bias associated with PCR. While it is prone to a higher raw read error rate associated largely with single insertions and deletions, the errors are random (not systematic as in earlier approaches) which can be resolved by the consensus step of the assembly and Illumina short reads polishing. Oxford nanopore sequencing is another approach to single-molecule sequencing (SMS) that has read length, error rate, and throughput similar to PacBio but is primarily available as a portable, cheap, real-time device called MinION that can be directly connected to a computer and conveniently used in the field. It does not depend on chemical labeling of the sample or intervening PCR amplification steps (Ambardar et al., 2016; Rang et al., 2018). Instead, the individual nucleotides are identified as a single DNA or RNA molecule is transported through a nanopore (nanometers in size) using electrophoresis. There also exist a number of other approaches based on the idea of direct imaging of the polynucleotides using tunneling and transmission electron microscopy (Schadt et al., 2010). One of the most important applications of TGS is its role in genome assembly and full-length transcripts identification due to its ultra long read length compared to NGS. This has resulted in significantly higher quality of genomes for an increasing number of species.

## Evolution of Transcript Profiling

Some of the earliest attempts at transcript profiling date back to the Sanger sequencing in 1980s, of the expressed sequence tags (ESTs), which are short nucleotide sequences generated from cDNAs (Adams et al., 1991; Marra et al., 1998). Other methods such as Northern blotting and reverse transcriptase quantitative PCR (RT-qPCR) were often used as *ad hoc* options for targeting few transcripts (Alwine et al., 1977; Becker-André and Hahlbrock, 1989; Morozova et al., 2009). The mid-1990s saw the rise of two different genomic scale approaches to transcript characterization, namely serial analysis of gene expression (SAGE) (Velculescu et al., 1995), and DNA microarrays (Lockhart et al., 1996). SAGE involves sequencing (initially Sanger sequencing) of long concatemers of small tags (initially ∼10 bp) that uniquely identify different mRNAs. A statistical analysis of the frequency of the tags and the corresponding mRNA sequences allows a direct transcript quantification and discovery of new genes. Over the years, variations of SAGE have been devised to identify tags more accurately by increasing tag length to 17 (LongSAGE, Saha et al., 2002), 21 (Robust-LongSAGE, Gowda et al., 2004), and 26 (SuperSAGE, Matsumura et al., 2005). Another variation led to massively parallel signature sequencing (MPSS) based on sequencing reads of 16–20 bp (Brenner et al., 2000), which was used to validate the expression of around 10,000 genes in *Arabidopsis thaliana* (Meyers et al., 2004) and similarly for around 20,000 genes across 32 human tissues (Jongeneel et al., 2005). DNA microarrays (or DNA chips) are based on the concept of measuring the hybridization of the labeled target cDNA strands from sample with the fixed probes (Schena et al., 1995). Because of their high throughput and lower cost, microarrays were widely used throughout 2000s. However, unlike SAGE, they are limited to probing using the array the genes that are already known, so a reference genome or transcriptome is a must for microarrays.

High throughput sequencing, beginning in the early 2000s, has sought to address the limitations inherent to previous approaches. More specifically, RNA-Seq supports both the discovery and quantification of transcripts using a single high-throughput sequencing assay. A reference genome or a transcriptome is used for read alignment but if a reference sequence is not available, a transcriptome can be assembled *de novo* using the reads and subsequently used for read alignment. Also, it allows quantification of RNAs over a broader dynamic range of five orders of magnitude, as compared to three for microarrays. In addition to gene expression quantification, RNA-Seq is quite effective in detecting alternative splicing events. As a result, it has grown to be most popular transcript profiling approach over the last decade. However, based on second generation sequencing approaches, the short-read RNA-Seq has several inherent limitations. It fails to accurately identify multiple full-length transcripts reconstituted from the short reads (Steijger et al., 2013; Wang et al., 2016). This problem is pervasive particularly when dealing with complex genomes (mostly eukaryotic), which exhibit a large number of isoforms per gene because of alternative splicing and where genes have multiple candidate promoters and 3′ ends (Conesa et al., 2016). As a result, short-reads RNA-Seq is simply insufficiently equipped in studying gene regulation, the protein-coding potential of the genome and ultimately the phenotypic diversity.

With long-read sequencing technologies, it has become reality that one read is one transcript, and each transcript can be accurately captured and studied individually since it directly provides full-length cDNA sequences (Wang et al., 2016). Techniques such as Oxford Nanopore and PacBio SMS, are designed to do away with the need to do assembly and therefore are better suited to comprehensively identify full length transcripts and to profile allele specific expression. While TGS techniques are optimal for *de novo* sequencing for small-to-moderate sized genomes (<1 Gbp), they become cost-prohibitive for high coverage of larger genomes. In such cases, a hybrid approach combining the strengths of SGS and TGS yields less erroneous outcomes at lower costs (Koren et al., 2012; Goodwin et al., 2015; Miller et al., 2017).

Unlike the previous approaches, single-molecule long-read sequencing based transcript profiling techniques have the inherent advantage of rendering, *in vitro* and without ambiguity, a full-length transcript sequence without depending on the error-prone, computational step of assembly (Abdel-Ghany et al., 2016; Wang et al., 2016; Cheng et al., 2017). As a result, they allow a more precise detection of alternative splicing events and eventually novel isoforms, making it easier to build gene models for species which are poorly studied or have an incomplete or missing reference genome. Next, we will discuss one of the most popular third generation transcript profiling techniques, namely, PacBio Iso-Seq.

## Pacbio Iso-Seq

Pacific Biosciences offers Iso-Seq protocol for transcript sequencing that includes library construction, cDNA fragment size selection, sequencing, and data analysis for characterization of multiple isoforms (Au et al., 2013; Tilgner et al., 2014, Wang et al., 2016). Here we briefly discuss the *in vitro* and *in silico* stages of the Iso-Seq protocol.

### Experimental Pipeline

**FIGURE 1:**
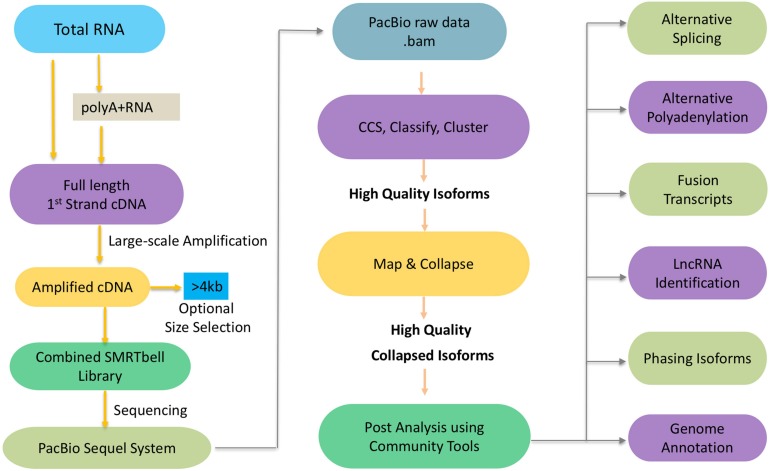
Schematic workflow of Iso-Seq.

To get the high-confidence transcripts set, we recommend to start the experimental pipeline with size selection (BluePippin and SageELF^TM^ Size Selection systems), which will result in libraries for multiple size fractions (e.g., <1 kb, 1–2 kb, 2–3 kb, 3–5 kb, and (>5 kb). Size selection is recommended to get the best out from your libraries since it allows a more accurate detection over a broader range of transcripts. In the absence of size selection, smaller fragments may load preferentially on the sequencer necessitating more SMRT cells in total since each library requires a certain number of cells to get sufficient depth to capture as many transcripts as possible. With the development of sequencing platform and chemistry, it is worth noting that the Sequel sequencing kit and protocols eliminate the need for size selection for transcripts < 4 kb but size selection can be optionally used to enrich for transcripts > 4 kb (Figure 1). While this has significantly streamlined the downstream steps in the experimental pipeline, it can potentially introduce sequencing bias for libraries that exhibit a large size range.

### Informatics Pipeline

Next we will discuss the informatics pipeline that leverages the sequencing reads from the experimental pipeline toward the goal of generating high quality isoforms *de novo* which may optionally be mapped to a reference genome (Figure 1). Here is a brief outline of the steps involved. The PacBio raw reads are continuous long reads (CLR) that need to be trimmed for adapters and filtered for artificial artifacts. Depending on lengths of the CLR and the transcript, the lifetime of the polymerase and the number of times an inserted strand was sequenced (number of passes), one or more subreads are generated. The subreads from a single ZMW are used to generate a circular consensus sequence (CCS) read. The reads are classified into full-length non-chimeric (FLNC), and non-FLNC reads. FLNC reads contain both 5′ and 3′ primers as well as a poly(A) tail preceding the 3′ primer. The FLNC reads are grouped into consensus isoforms using iterative clustering for error (ICE) correction. At this stage, tools such as Quiver (Chin et al., 2013) can be used to incorporate non-FLNC reads to polish the consensus isoforms and select the high quality isoforms. Also, short reads from RNA-Seq if available can be used for an additional step of error correction using tools such as LoRDEC (Salmela and Rivals, 2014), LSC (Au et al., 2012), or Proovread (Hackl et al., 2014). If a reference genome is available, these high quality isoforms can be mapped against it using tools such as GMAP (Wu and Watanabe, 2005), minimap2 (Li, 2018), and STAR (Dobin et al., 2013). The mapped transcripts can be collapsed further to filter out redundant transcripts using ToFU (Gordon et al., 2015) or TAPIS (Abdel-Ghany et al., 2016). PacBio SMRT Link Suite offers various command line and programmatic options as well as a web-based user interface to support analysis and end-to-end workflow management of the reads from PacBio RS II and Sequel systems (PacBio GitHub^1^).

An improved version of the pipeline, Iso-Seq2, has an extra pre-clustering step to bin full length non-chimeric reads based on gene families. The subsequent steps are similar to Iso-Seq1. The latest version of the pipeline, Iso-Seq3, is designed to scale up to the much higher throughput of Sequel compared to PacBio RS II because of optimization features such as faster clustering algorithms. Also, the Iso-Seq3 pipeline generates relatively fewer but higher quality polished transcripts than Iso-Seq2 because of a more conservative primer removal and barcode demultiplexing step (named, lima). Unlike the previous versions, it also does away the need to use non-full reads. A quality check using SQANTI (Tardaguila et al., 2018) also confirms that Iso-Seq3 generates a higher number of perfectly annotated isoforms. Please see Table 1 for a listing of the Iso-Seq tools discussed in this manuscript.

**TABLE 1:** List of the Iso-Seq tools along with a brief description of their usage and related online links.

Tool	Usage	Website	Literature
ASTALAVISTA	Detect alternative splicing events	http://astalavista.sammeth.net/	Foissac and Sammeth,2007
CASH	Detect alternative splicing events	https://sourceforge.net/projects/cash-program/	Wu et al.,2018
CodingQuarry	Gene prediction (HMM-based) using both RNA-Seq data and genome sequence	https://sourceforge.net/projects/codingquarry/	Testa et al.,2015
GMAP	Spliced alignment to genome	http://research-pub.gene.com/gmap/	Wu and Watanabe,2005
LoRDEC	Error correction of FLNC with short read RNA-seq	http://atgc.lirmm.fr/lordec	Salmela and Rivals,2014
LoReAn	Comparative analysis and annotation: identify novel isoforms/genes against reference annotation	https://github.com/lfaino/LoReAn	Cook et al.,2018
LSC	Error correction of FLNC with short read RNA-seq	http://augroup.org/LSC/LSC_download.html	Au et al.,2012
minimap2	Spliced alignment to genome	https://github.com/lh3/minimap2	Li,2018
PASA	Detect alternative splicing events	https://pasapipeline.github.io/	Liu et al.,2017
Proovread	Error correction of FLNC with short read RNA-seq	https://github.com/BioInf-Wuerzburg/proovread	Hackl et al.,2014
Quiver	Polishing PacBio RS II reads	https://github.com/PacificBiosciences/ GenomicConsensus	Chin et al.,2013
SpliceGrapher	Detect alternative splicing events	http://splicegrapher.sourceforge.net/	Rogers et al.,2012
SQANTI	Comparative analysis and annotation: identify novel isoforms/genes against reference annotation	https://bitbucket.org/ConesaLab/sqanti	Tardaguila et al.,2018
STAR	Spliced alignment to genome	https://github.com/alexdobin/STAR/releases	Dobin et al.,2013
SUPPA	Detect alternative Splicing events	https://bitbucket.org/regulatorygenomicsupf/suppa	Alamancos et al.,2015
TAPIS	Alternative splicing, collapsing redundant or degraded transcripts	https://bitbucket.org/comp_bio/tapis	Abdel-Ghany et al.,2016
ToFU	Preprocessing (collapse to non-redundant isoforms)	https://github.com/PacificBiosciences/IsoSeq_SA3nUP	Gordon et al.,2015

## Downstream Applications of Iso-Seq

In addition of the discovery of novel transcripts and alternative splicing events, the availability of high quality, full-length isoform sequences greatly impacts our understanding of alternative splicing, alternative polyadenylation (APA), fusion transcripts, long non-coding RNAs (lncRNAs), isoform phasing, and genome annotation (Figure 1).

### Identification of Alternative Splicing

Alternative splicing is one of the most common mechanisms known to increase the diversity of transcripts primarily in eukaryotes. Before the advent of TGS, the traditional method to identify different splicing isoforms has been based on the short-reads sequencing, which assembles short reads into long transcripts based on splice junction reads. This approach often results in prediction of transcripts that do not exist (false positives) or fails to identify true transcripts (false negatives), especially when one gene can transcribe a large number of isoforms. With the development of SMS technology, “one read is one transcript” is not a dream anymore and scientists can get the intact sequence of each isoform by sequencing a single cDNA molecule. Since no assembly is required in this method, it eliminates the assembly errors caused by previous short-reads sequencing and offers particular advantage in characterization of polyploid transcriptomes which have a large number of repeats and homeolog genes. There are a number of different events that can lead to alternative splicing: exon skipping (ES), alternative 5′ splice site (A5), alternative 3′ splice site (A3), mutually exclusive exons (MXE), and intron retention (IR). Tools that detect alternative splicing events include Astalavista (Foissac and Sammeth, 2007), SUPPA (Alamancos et al., 2015), PASA (Program to Assemble Spliced Alignments) (Liu et al., 2017), SpliceGrapher (Rogers et al., 2012), and CASH (Wu et al., 2018). Compared to SGS based tools such as reference-guided (Cufflinks, StringTie) or *de novo* (Trinity, Oases, Velvet), Iso-Seq is known to retrieve longer isoforms as well as more number of isoforms (both total and per gene) (Gordon et al., 2015; Wang et al., 2016). This has revolutionized our understanding of the biology of a number of organisms, including plants and animals since transcript diversity usually represents functional diversity, indicating the potential important biological functions of these novel identified isoforms (Au et al., 2013; Abdel-Ghany et al., 2016; Wang et al., 2016, 2018;Kuo et al., 2017).

### Identification of Alternative Polyadenylation

In addition to the APA is another widespread mechanism in complex genomes, particularly eukaryotic, for post-transcriptional regulation of function, stability, localization, and translation efficiency (Shen et al., 2008, 2011). Alternative polyadenylation controls gene expression by virtue of selection of alternate poly(A) sites in the 3′ end of the pre-mRNA, thus letting a gene encode multiple mRNA transcripts which vary in their coding sequence (CDS) or often in their 3′UTR regions. While normally, it is found in the distal region of 3′ UTR, it has number of other variations including proximal region of 3′ UTR, alternative terminal exons, intronic sites, and exonic CDS sites (Gruber et al., 2013). Once again, while it’s challenging to detect alternative polyadenylation sites using short reads from SGS, full-length cDNA sequencing from Iso-Seq is able to detect genome-wide alternative polyadenylation sites, and the 3′ end is more accurate because of the poly(A) selection during the library construction. As a result, alternative polyadenylation motif has been identified from different species (Abdel-Ghany et al., 2016; Wang et al., 2018).

### Fusion Transcript Identification

A fusion transcript is a chimeric RNA made of two or more transcripts. Often, the constituent transcripts correspond to two distinct genes brought together into a fusion gene at DNA level because of translocation, interstitial deletion, or inversion. Alternatively, transcripts can fuse at RNA level by the *trans-*splicing or *cis-*splicing between the neighboring genes (Kumar et al., 2016). The constituent transcripts must map to two or more loci which are at least 100 kb apart, align at least 10% with the corresponding transcripts and together contribute to at least 99% alignment coverage (Wang et al., 2016). While there exist dozens of SGS tools that can detect fusion transcripts, they are limited because of mapping errors inherent to short reads and the assembly. The Cupcake ToFu (Gordon et al., 2015) developed by PacBio has been able to identify candidate fusion transcripts, and another tool is Isoform Detection and Prediction (IDP-fusion) which uses a hybrid approach based on SGS and TGS reads and was able to identify fusion genes and their isoforms in cancer transcriptomes (Weirather et al., 2015). However, those candidate fusion transcripts usually have high false positive rate which need further validation through different approaches, e.g., RT-PCR followed by Sanger sequencing or single-molecule mRNA Fluorescent *in situ* Hybridization (RNA FISH) (Semrau et al., 2014; Wang et al., 2016).

### Single-Molecule Sequencing Facilitates Genome Annotation

Many of the commonly used annotation pipelines use a combination of *ab initio* and evidence based predictions to generate accurate consensus annotations. MAKER2 is a user-friendly, fully automated annotation pipeline that incorporates multiple sources of gene prediction information and has been extensively used to annotate eukaryotic genomes (Holt and Yandell, 2011). The Broad Institute Eukaryotic Genome Annotation Pipeline (Haas et al., 2011) has mainly been used to annotate fungal genomes and integrates multiple programs and evidences for genome annotation. CodingQuarry (Testa et al., 2015) is another gene prediction software that utilizes general hidden Markov models for gene prediction using both RNA-Seq data and genome sequence. However, most of these tools are not designed to exploit gene structure information from single-molecule cDNA sequencing.

The use of single-molecule cDNA sequencing can increase the accuracy of automated genome annotation by improving genome mapping of sequencing data, correctly identifying intron exon boundaries, directly identifying alternatively spliced transcripts, identifying transcription start and end sites, and providing precise strand orientation to single exons genes. The full-length transcripts mapped against a reference genome can be used to improve or add *de novo* structural and functional annotation to a genome, improve genome assembly and existing gene models. Previous studies have demonstrated the advantage of SMS by discovering longer and novel transcripts/genes, lncRNAs, and even fusion transcripts as well (Abdel-Ghany et al., 2016; Wang et al., 2016). To address the disconnection between genome annotations and the latest sequencing technologies, recently, the Long Read Annotation (LoReAn) pipeline has been developed (Cook et al., 2018). LoReAn is an automated annotation pipeline that takes full advantage of MinION or PacBio SMRT long-read sequencing data in combination with protein evidence and *ab initio* gene predictions for full genome annotation. Short-read RNA-Seq can be used in LoReAn to train *ab initio* software. Based on the reannotation of two fungal and two plant species, LoReAn has been shown to provide annotations with increased accuracy by incorporating single-molecule cDNA sequencing data from different sequencing platforms. SQANTI (Tardaguila et al., 2018) is another pipeline for structural and quality annotation of novel transcript isoforms. It takes as input the full length transcripts and a reference genome and associated annotations, and provides a deep characterization of isoforms at both transcript and junction level. It generates gene models and classifies transcripts based on splice junctions and donor and acceptor sites. In addition, it can also filter out isoforms that are likely to be artifacts.

### Single-Molecule Sequencing Enables Isoform Phasing

Haplotype phasing of genetic variants is important for interpretation of the genome, population genetic analysis, and functional genomic analysis of allelic activity. Even though more and more long-read sequencing reads have been generated for different studies, there is not much investigation on the allelic variants so far. Such information is crucial for understanding allelic transcriptomes, the parent origin of each allele, and their potential biological consequences. SMS has been used successfully to identify full-length gene isoforms and thus have the potential to overcome the haplotyping problem due to its multi-kilobase reads length. Recently, a series of tools have been developed for the haplotyping of single-molecule isoforms. IDP-ASE was developed for haplotyping and quantification of Allele-specific expression (ASE) at both the gene and isoform levels requiring only RNA sequencing data (Deonovic et al., 2017). HapIso is another method for the reconstruction of the haplotype specific isoforms of a diploid cell, which is able to tolerate the relatively high error-rate of the SMS and discriminate the reads into the paternal alleles of the isoform transcript (Mangul et al., 2017). phASER (Castel et al., 2016), was developed to incorporate RNA-Seq and DNA-Seq data with population phasing, allowing phasing over longer distances. And IsoPhase, which is under development from PacBio, is designed to phase the isoforms from diploid or even tetraploid organisms. With IsoPhase, parent-of-origin allele specific isoforms can be identified in the hybrids. Firstly CCS reads are aligned to genome, then individual SNPs are called, and full length reads are used to infer haplotypes, residual sequencing errors are corrected to get to the number of expected alleles, finally the number of full-length reads of each haplotype can be called.

## Spatio-Temporal Variability in Transcriptome Profile

**FIGURE 2:**
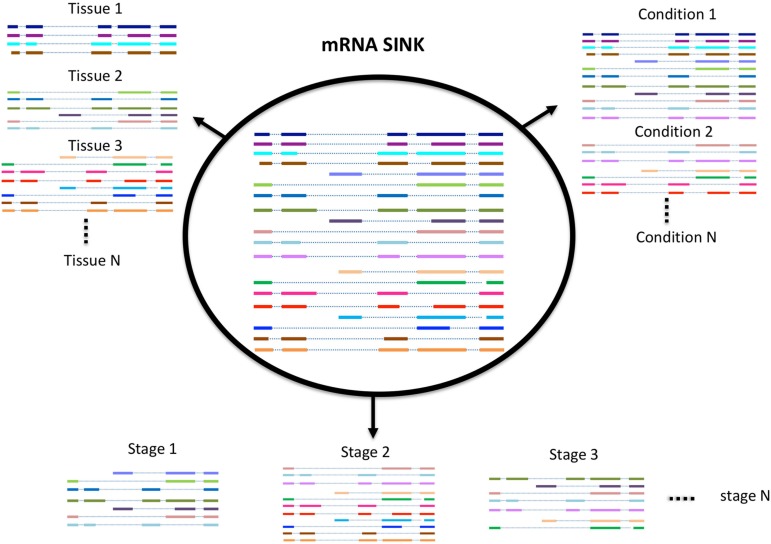
Schematic model of alternative splicing utilization.

While Iso-Seq has been successful in identifying a large number of novel and longer transcripts in almost all species where it was used, most of these transcripts lack an experimental or evidence-based functional characterization. A number of studies exist that have demonstrated that the number of transcripts expressed in an organism (transcriptome profile) depends on many factors such as environmental stress, growth condition, developmental stage, and tissue type (Figure 2; Wang et al., 2016, 2018; Zhu F.Y. et al., 2017). Therefore, the diversity of transcripts in one organism can be increased with the sequencing of more and more tissues. Previous approaches mostly use short reads sequencing to identify potential transcripts in a certain tissue, this approach is good to quantify the expression level of each transcript, but not able to give the accurate information or complete structure of the transcript. In contrast, SMS due to its ultra long reads methodology is significantly more accurate. Recently, it has also become feasible to study the full-length transcriptome at single cell level both in animals and in plants (Zhu S. et al., 2017; Ryu et al., 2019). We believe with the development of new techniques and participation from more labs, the diversity of transcriptome within or between species will be further revealed.

## How to Deal With Multiple Isoforms Identified From Iso-Seq?

**FIGURE 3:**
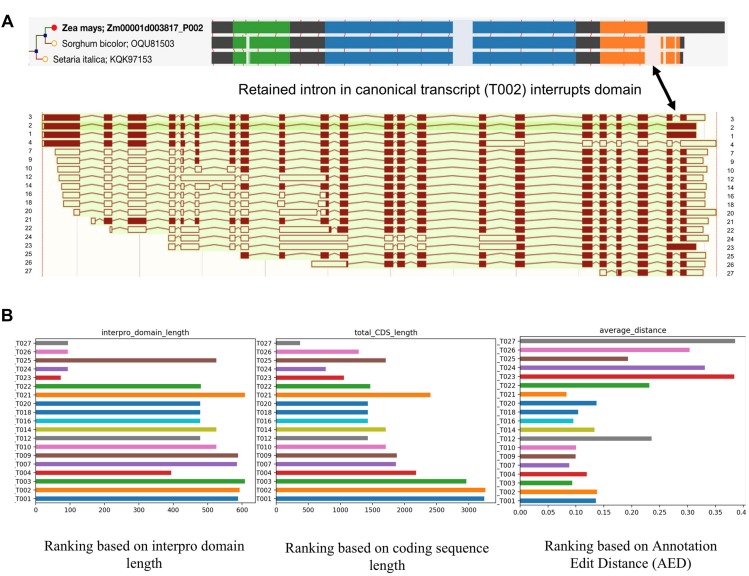
Alignment and ranking of different isoforms. **(A)** Gene tree multiple sequence alignment color coded by interpro domain. The orange domain is conserved in grass orthologs, but is not identified completely in maize because a retained intron disrupts it and induces a frame shift (shown by arrow). The resulting longer translation was selected for analysis in the gene tree pipeline. Different colors mean different interpro domains, black color means “no domain here,” lightly shaded area indicates a gap in the multiple sequence alignment. The thin red lines show the positions of exon junctions. **(B)** Ranking of different isoforms of Zm00001d003817 based on different standards. T002 has the longest CDS, but T003 outperforms it in domain length and annotation edit distance.

While single-molecule long read sequencing based approaches have identified a wide array of novel transcripts which were generated from different splicing patterns (Figure 3A), they need to be validated and characterized since not all of them have a meaningful impact on the cellular biological processes of the cell. Recent studies in maize and sorghum (Wang et al., 2018) showed that ∼45% of the isoforms could undergo Non-Sense Mediated Decay (NMD) after mRNA processing; that being said, a large number of the transcripts potentially will be degraded before transportation to the cell and the rest of transcripts are more likely to have biological functions. Therefore, there is clearly a need to be able to judge the validity and usage of these isoforms. We propose that high confidence transcripts can be ranked for validity based on criteria such as open reading frame (ORF) and CDS length, Interpro domain coverage, annotation edit distance, and their spatio-temporal expression levels. Figure 3B illustrates the application of such criteria to an example gene Zm00001d003817 from maize. The result showed that the ranking of isoforms can be different using different criteria. Due to an IR in T002 isoform, its ORF was shifted, and as a result the protein domain which is conserved in grass orthologs is not completely identified in maize. T002 has the longest CDS, but T003 outperforms it in domain length and annotation edit distance.

## Comparative Single-Molecule Transcriptome Studies Between Close Species, What to Compare?

A growing number of SMS based transcriptome studies have made it possible to compare full-length transcriptomes between evolutionarily close species and identify the cause of divergence of different phenotypes between species. Based on the orthologous genes in the two organisms and the associated full-length transcriptomes, we can now compare the splicing variants between species and better understand the conservation of genes/isoforms, the divergence of splicing patterns, and the significance of their expression levels. The first SMS based comparative transcriptome study was performed between maize and sorghum by Wang et al. (2018). Conserved genes and isoforms were identified between these two species, gene expression and alternative splicing were found to be playing an important role in the plant architecture divergence between evolutionarily close species. However, certain requirements are needed to perform these comparative studies, such as: (1) tissues selected in this study should be at same or very similar developmental stage for the comparison to be meaningful; (2) there should exist a threshold depth of sequencing, so that majority of isoforms will be captured in each tissue/organism.

## Conclusion

It is worth noting that as the TGS platforms continue to mature, they are not without their own set of challenges. Three of the more common challenges associated especially with the early PacBio long reads are the raw read errors, low throughput and high cost. Higher than acceptable errors in raw reads limit the *de novo* transcript identifications, necessitating the need for the reference genome (Au et al., 2012). Low throughput from SMRT cells makes it difficult to accurately quantify the transcript expression. As a result, most of the captured isoforms are highly expressed isoforms and the lowly expressed isoforms are usually lost. Also relatively longer transcripts are more likely to be missed due to longer polymerase lifetime required to allow full-length pass during the sequencing. That being said, sequencing depth matters for Iso-Seq study, especially when it comes to comparison between different tissues or conditions, or even different species, therefore a higher sequencing depth is necessary to make the comparison convincing. As a gap-fill measure, the long read dataset can be supplemented with more accurate and abundant short reads, if available, to address these issues (Hansen et al., 2011). PacBio Sequel, with its improved chemistry, tries to address these concerns by offering higher sequencing lengths amenable to more number of passes for consensus auto-correction as well as higher throughput from SMRT cells.

With the reality that Iso-Seq transcripts have been used to annotate more and more genomes, another challenge is the need to rank and prioritize for community research the growing number of isoforms identified from different tissues/conditions within an organism. While SMS has dominated the transcriptome sequencing with its power of identification of full-length information of each transcript, it has raised new questions such as, how to deal with the large number of newly identified isoforms and what are their functions. Experimental approaches such as CRISPR could help by targeting the role of each isoform, and see if there are redundant or complementary functions among these different splicing isoforms.

## Author Contributions

BW and VK developed the conceptual outline and drafted the manuscript. BW, VK and AO contributed figures and a table. All authors contributed to reviewing the final manuscript.

## Conflict of Interest Statement

The authors declare that the research was conducted in the absence of any commercial or financial relationships that could be construed as a potential conflict of interest.

## Footnotes

1 https://github.com/PacificBiosciences/pbcommand
